# Supplementary material for: Land-cover change in Cuba and implications for the area of distribution of a specialist’s host-plant
Source: PeerJ. 2024 Jun 25;12:e17563. doi: 10.7717/peerj.17563 (PMC11212621; doi:10.7717/peerj.17563)
Supplement: Supplemental Information 1 [file peerj-12-17563-s001.docx]

**Supplementary materials for the manuscript “Land-cover change in Cuba and how it is affecting the areas of distribution of *Omphalea* (Angiosperma: Euphorbiaceae) and *Urania boisduvalii* (Lepidoptera: Uraniidae)”**

Claudia Nuñez-Penichet^1*^, Juan Maita-Chamba^2^, and Jorge Soberón^1^

^1^Biodiversity Institute and Department of Ecology & Evolutionary Biology, University of Kansas.

^2^Carrera de Ingeniería Forestal, Centro de Investigaciones Tropicales del Ambiente y Biodiversidad, Universidad Nacional de Loja, Loja 110111, Ecuador

^*^Corresponding Author: Claudia Nuñez-Penichet

Biodiversity Institute and Department of Ecology & Evolutionary Biology, University of Kansas, 1345 Jayhawk Blvd., Lawrence, Kansas 66045, USA. [claununez199o@gmail.com](mailto:claununez199o@gmail.com)

S1 Table. Values of the overall accuracy of the classification, for the years 1985 and 2020, for each land cover type considered in this study.

| Cover type | Overall accuracy (%) | |
| --- | --- | --- |
|  | 1985 | 2020 |
| Forest and shrubs | 98 | 99 |
| Mangrove | 91 | 95 |
| Soil without vegetation cover | 83 | 89 |
| Wetlands | 92 | 71 |
| Pine forest | 80 | 72 |
| Agriculture | 100 | 95 |
| Water bodies | 97 | 98 |

S1 Figure. Vegetation types in the areas of potential distribution of *Omphalea* plants in Cuba for the years 1985 and 2020. *Omphalea* includes all the species of *Omphalea* genus distributed in Cuba combined.


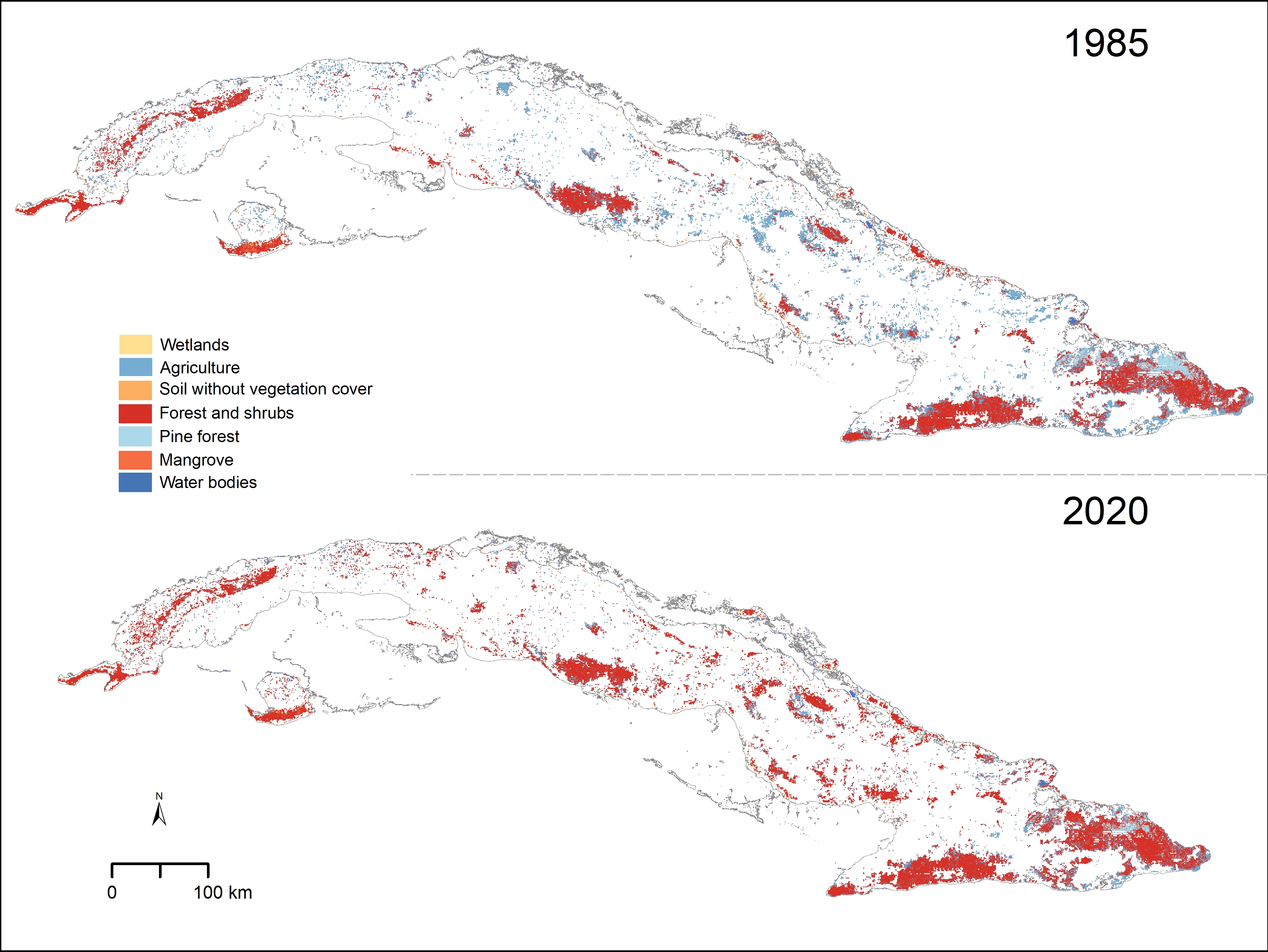


S2 Figure. Vegetation types in the terrestrial protected areas of Cuba for the years 1985 and 2020.


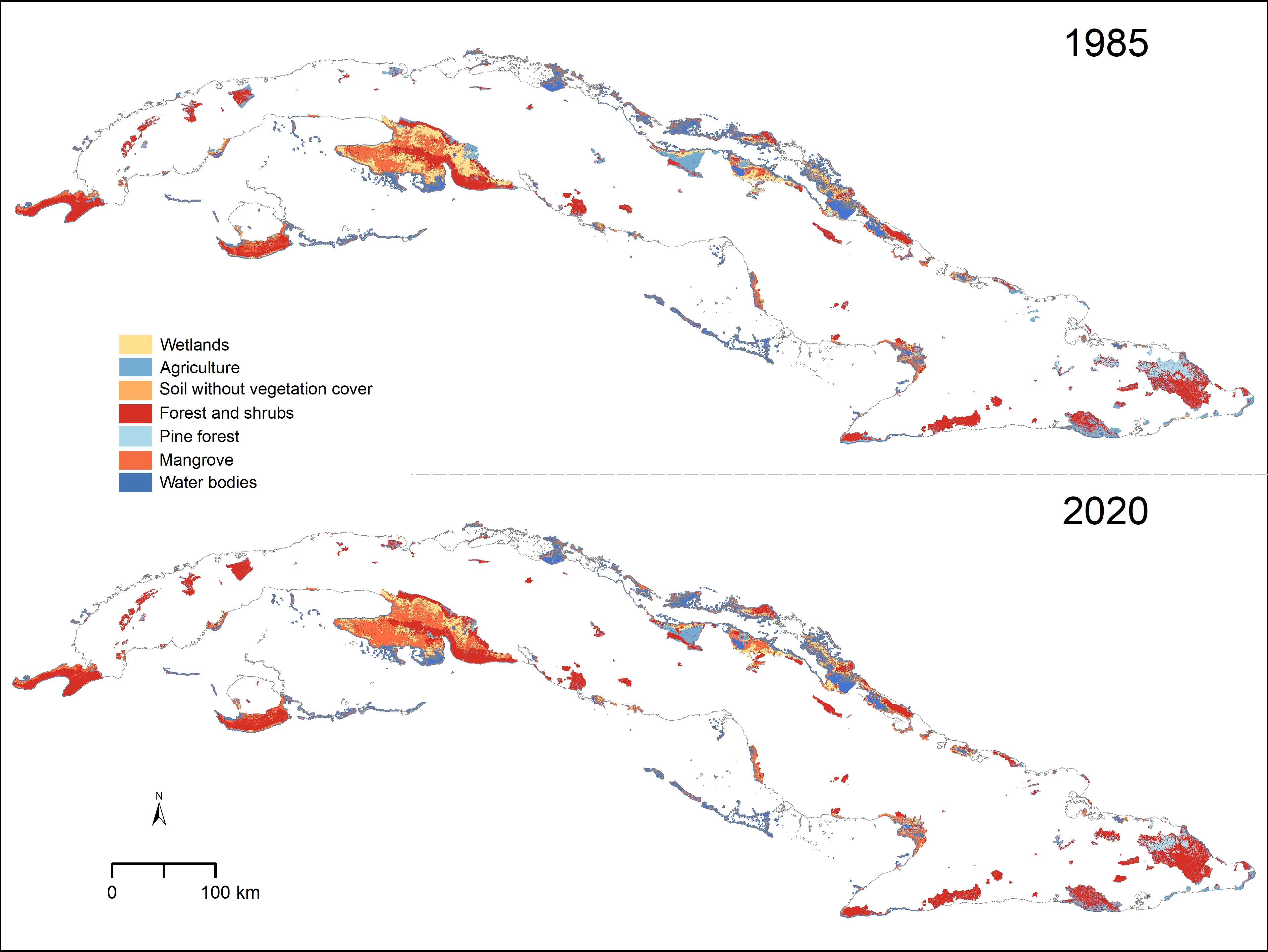


S3 Figure. Cover types in the areas of potential distribution of *Omphalea* plants in Cuba that are inside the terrestrial protected areas. *Omphalea* includes all the species of *Omphalea* genus distributed in Cuba combined and the areas of potential distribution of *Omphalea* is referring to these species’ potential distribution in Cuba.


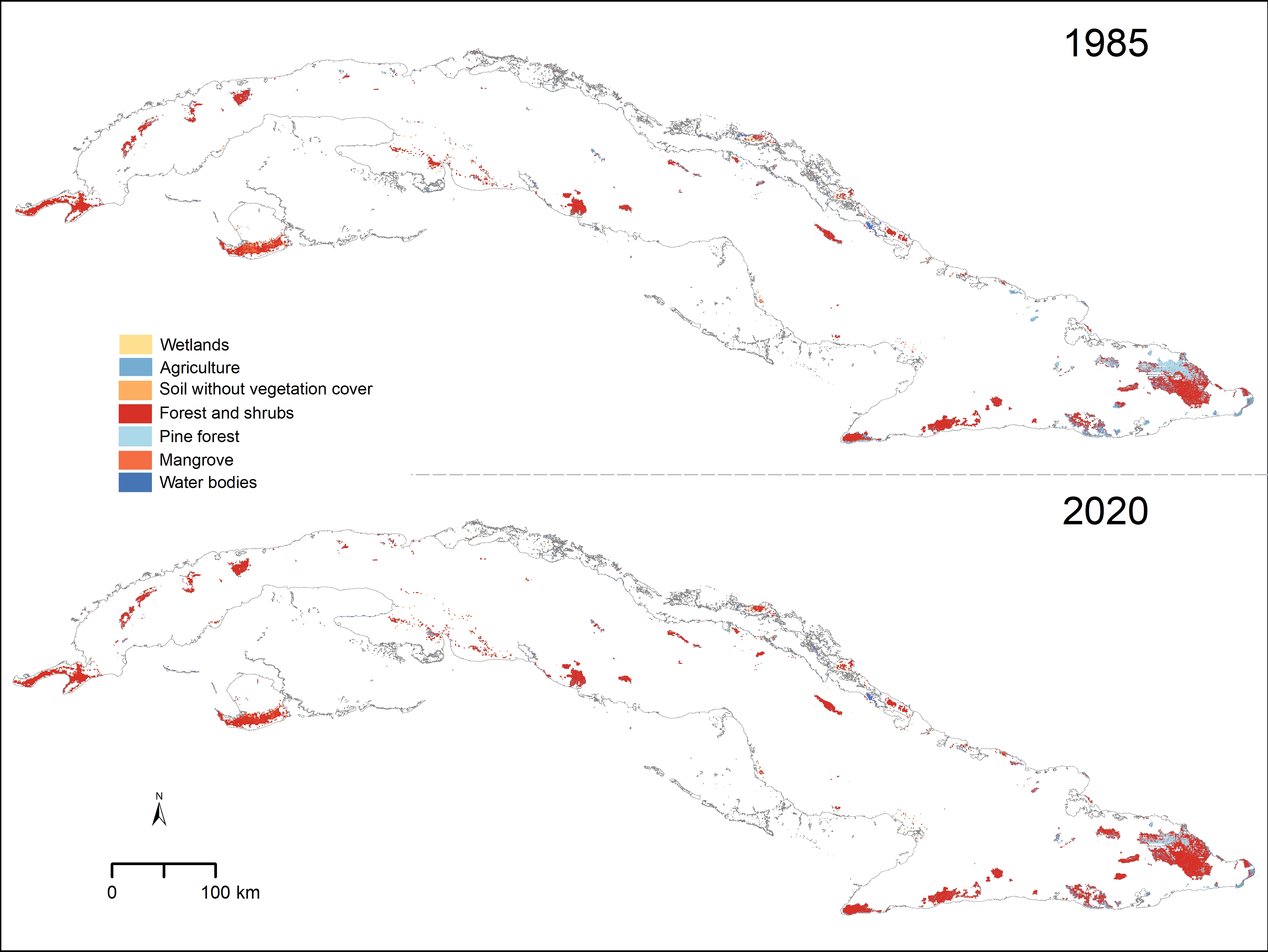


S1 Appendix 1. Description of the area of study.

Cuban archipelago is located on the northeast side of the Caribbean Sea (-84.993 – -74.111 longitude, 19.837 – 23.233 latitude), is part of the Greater Antilles, and has a surface of approximately 110922 km2 (Gutiérrez and Rivero, 1997; Fig. 1). Cuba’s landscape is formed mostly by lowlands with few elevations (Díaz, 1989; Fig. 1). The neotropical location of this archipelago, which determine its temperature (15 °C – 27 °C mean annual) and precipitation (736 ml – 2879 ml mean annual), together with the tectonic movements and volcanic activity from the past (Formel, 1989), influence the soil types of Cuba as well as its vegetation (Gutiérrez and Rivero, 1997).


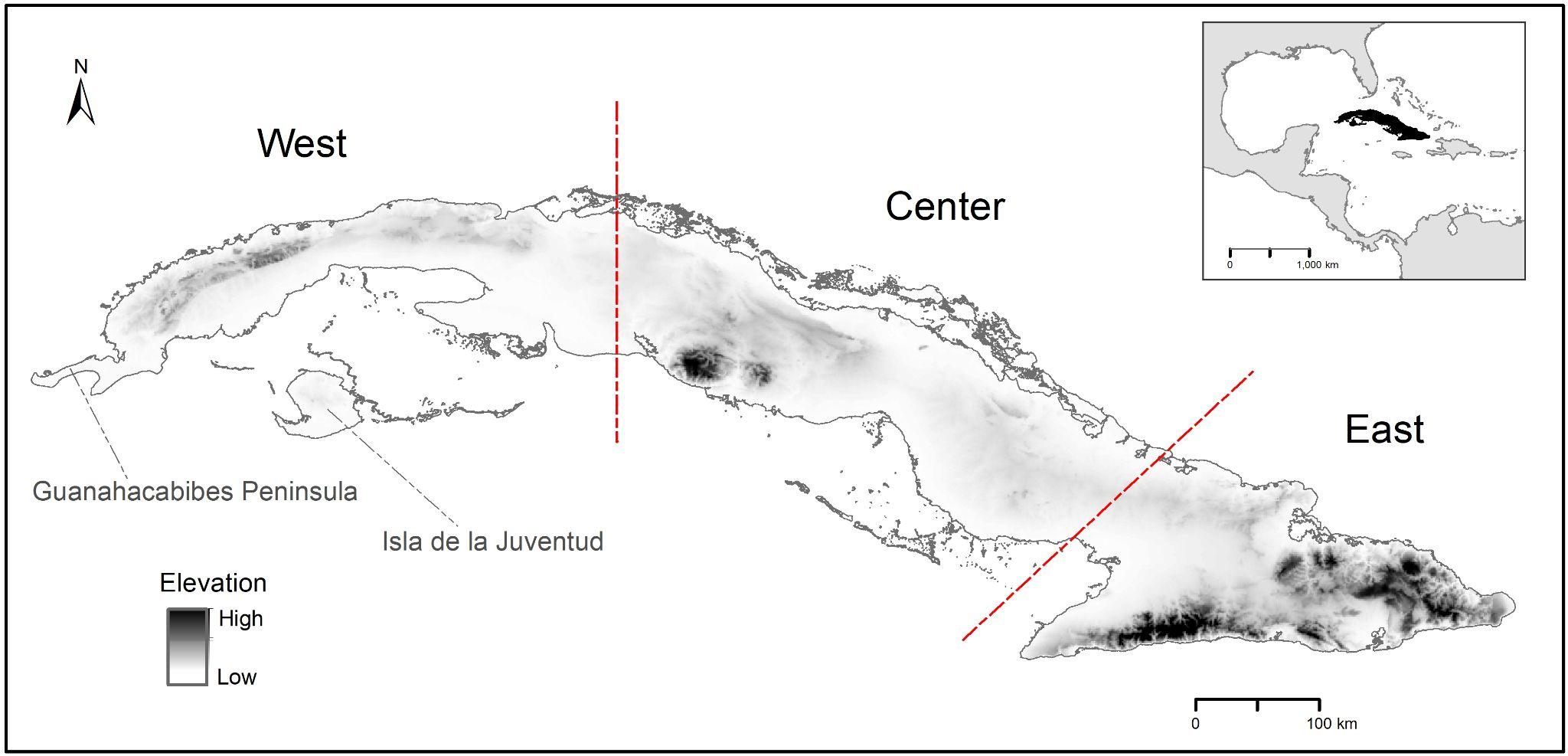


Figure 1. Study area.

References

Díaz, L.R., 1989. Nuevo atlas nacional de Cuba. Instituto Cubano de la Academia de Ciencias de Cuba, La Habana, Cuba.

Formel, F.C., 1989. Nuevo atlas nacional de Cuba. Instituto cubano de la Academia de Ciencias de Cuba, La Habana, Cuba.

Gutiérrez, R.D., Rivero, M., 1997. Minigeografía de Cuba. Editorial Científico-Técnica, La Habana, Cuba.

S1 Appendix 2. Elevational considerations used for post-processing the classified images to correct misclassified pixels. We used a raster calculator and a DEM of 30 m of resolution.

**Reclassification of pixels on 1985 land cover map using elevational considerations**

1. Mangrove pixels ⋝ 20 m above sea level change to pine forest
2. Wetland pixels ⋝ 11 m above sea level change to agriculture
3. Pine forest pixels ⋜ 20 m above sea level change to mangrove
4. Agriculture land pixels ⋜ 4 m above sea level change to wetlands
5. Water bodies pixels ⋝ 500 m above sea level change to forest and shrubs

**Reclassification of pixels on 2020 land cover map using elevational considerations**

1. Mangrove pixels ⋝ 20 m above sea level change to pine forest
2. Wetland pixels ⋝ 11 m above sea level change to agriculture
3. Pine forest pixels ⋜ 20 m above sea level change to mangroves
4. Agriculture land pixels ⋜ 4 m above sea level change to wetlands
5. Water bodies pixels ⋝ 500 m above sea level change to forest and shrubs
